# Continuous EEG monitoring by a new simplified wireless headset in intensive care unit

**DOI:** 10.1186/s12871-020-01213-5

**Published:** 2020-12-07

**Authors:** Anselmo Caricato, Giacomo Della Marca, Eleonora Ioannoni, Serena Silva, Tiziana Benzi Markushi, Eleonora Stival, Daniele Guerino Biasucci, Nicola Montano, Camilla Gelormini, Isabella Melchionda

**Affiliations:** 1 Department of Anesthesia and Intensive Care, Catholic University School of Medicine, Largo F. Vito, 1, 00168 Rome, Italy; 2 Neurosurgical Intensive Care, Fondazione Policlinico Universitario “A. Gemelli” IRCCS, Rome, Italy; 3 Stroke Unit, Fondazione Policlinico Universitario “A. Gemelli” IRCCS, Rome, Italy; 4 Department of Neurology, Università Cattolica del Sacro Cuore, Rome, Italy; 5 Department of Neurosurgery, Università Cattolica del Sacro Cuore, Rome, Italy

**Keywords:** Electroencephalography, Seizures, Critical care, Continuous EEG, NeuroIntensive care

## Abstract

**Background:**

In critically ill patients continuous EEG (cEEG) is recommended in several conditions. Recently, a new wireless EEG headset (CerebAir®,Nihon-Kohden) is available. It has 8 electrodes, and its positioning seems to be easier than conventional systems.

Aim of this study was to evaluate the feasibility of this device for cEEG monitoring, if positioned by ICU physician.

**Methods:**

Neurological patients were divided in two groups according with the admission to Neuro-ICU (Study-group:20 patients) or General-ICU (Control-group:20 patients). In Study group, cEEG was recorded by CerebAir® assembled by an ICU physician, while in Control group a simplified 8-electrodes-EEG recording positioned by an EEG technician was performed.

**Results:**

Time for electrodes applying was shorter in Study-group than in Control-group: 6.2 ± 1.1′ vs 10.4 ± 2.3′; *p* < 0.0001. Thirty five interventions were necessary to correct artifacts in Study-group and 11 in Control-group. EEG abnormalities with or without epileptic meaning were respectively 7(35%) and 7(35%) in Study-group, and 5(25%) and 9(45%) in Control-group;*p* > 0.05. In Study-group, cEEG was interrupted for risk of skin lesions in 4 cases after 52 ± 4 h. cEEG was obtained without EEG technician in all cases in Study-group; quality of EEG was similar.

**Conclusions:**

Although several limitations should be considered, this simplified EEG system could be feasible even if EEG technician was not present. It was faster to position if compared with standard techniques, and can be used for continuous EEG monitoring. It could be very useful as part of diagnostic process in an emergency setting.

## Background

Electroencephalogram (EEG) is a registration of cerebral electrical activity of the brain. It is conventionally performed by placing 20 electrodes on the scalp to detect excitatory and inhibitory postsynaptic potentials in neuronal dendrites, particularly in the most superficial regions of the cerebral cortex. Its recording usually lasts 20–30 min, and it is indicated in diagnosis of epileptic seizures, in differential diagnosis of movements disorders, in coma of unknown origin, as adjunctive test for brain death.

In critically ill patients, continuous EEG recording (cEEG) has been suggested. Recently, two consensus statements recommended this technique in several conditions: for diagnosis and the assessment of the therapy in non-convulsive seizures, in patients with unexplained and persistent altered consciousness, to assess cerebral ischemia, to monitor sedation, to assess the severity of encephalopathy and to improve prognostication of coma after cardiac arrest [1, 2].

American Society of Clinical Neurophysiology Guidelines specifically state that standard cEEG requires a minimum of 16 electrodes placed according with 10–20 International System, with placement designed to optimize brain regions sampled. If fewer than 16 electrodes are used, interpretation may be limited, and sensitivity for seizures may be low. Furthermore, recordings must be performed by appropriately trained, certified and supervised neurodiagnostic technologists [3].

Actually, this may be difficult to obtain in Emergency Department or in Intensive Care Unit, where logistic problems can be prevalent, and neurophysiologist can be not available. In this setting, EEG recordings could be not possible or limited to a short period with very low diagnostic power.

For this reason, simplified systems are now available; if they can be useful as emergency EEG is still not known.

Recently, a new headset (Cereb Air®, AE 120 A, Nihon Kohden Europe, Rosbach, Germany) has been proposed for its use in Intensive Care Unit (ICU). It has 8 electrodes, connects wireless to an electroencephalographer for digital recording, and its positioning could be easier and faster than conventional 10–20 system. It is used in 10-beds Neurosurgical Intensive Care Unit of “Fondazione IRCCS Policlinico Universitario “A. Gemelli” Hospital from 1st June 2017.

Primary aim of this single-center prospective observational study is to evaluate the feasibility of this EEG headset for cEEG monitoring in an emergency setting, if positioned by ICU physician.

## Methods

After signed informed consent obtained from relatives, each patient with subarachnoid hemorrhage, cerebral parenchymal hemorrhage or head injury and indication to cEEG, according with neurologist consultation, was consecutively included in the study. Surgical dressing that prevented the placement of EEG electrodes was considered as exclusion criterion. The study was approved by the Institutional Ethical Committee. Four topics were investigated: time for a correct positioning of electrodes, length of recording, number of interventions to correct artifacts, side effects.

In our hospital, neurological patients can be admitted to General ICU, if beds are not available in Neuro ICU. Thus, neurological patients were divided in two groups according with the admission to Neuro ICU (Study group) or General ICU (Control group). Twenty eight patients were screened. Three patients in study group and five in control group presented exclusion criteria; 20 patients in Study group and 20 patients in Control group were included in the final analysis. In Study group, EEG was recorded by the headset Cereb Air® assembled by a neuro ICU physician; Control group was studied in General ICU, where Cereb Air was not available, and a conventional simplified 8 electrodes EEG recording was assembled by an EEG technician.

**Fig. 1:**
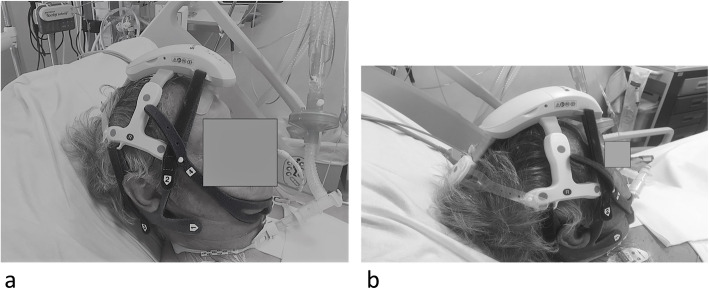
**a**, **b** The headset. Frontal and lateral view. Headset during EEG monitoring is shown. Black circles correspond to the position of the temporal and central frontal electrodes. In **b** the position of the occipital electrode can be observed

**Fig. 2:**
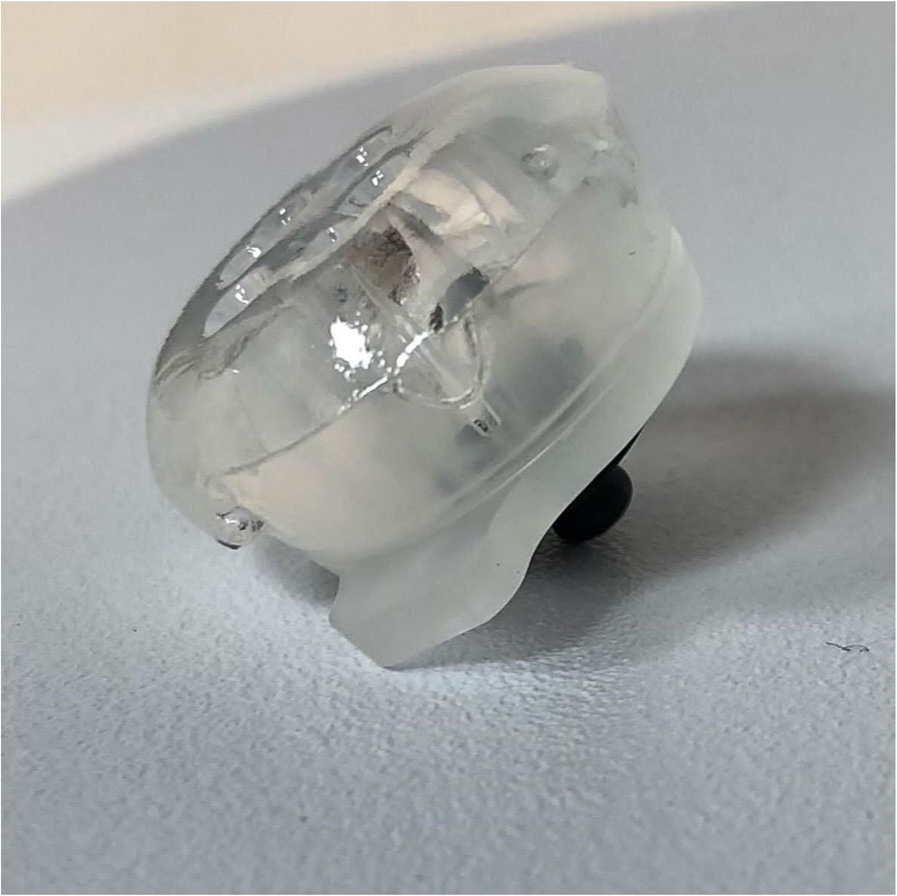
Gel electrode. Single-use gel electrode is shown

**Fig. 3:**
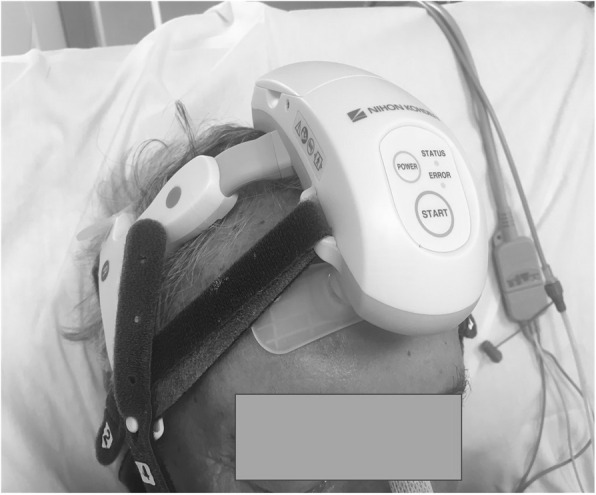
Body of the helmet. It contains batteries, two buttons for start and Bluetooth connection, and two lights

We used a wireless headset, (CerebAir® Nihon-Kohden) that is a plastic adjustable structure adaptable to the size of the patient’s head (Fig. 1a,b); 12 EEG tracings were obtained by 7 pre-constituted single-use electrodes (Fig. 2), which engages in defined points of the helmet, and a reference adhesive electrode (Z). It connects via a Bluetooth wireless system to the electroencephalographer (Fig. 3).

EEG recordings were reviewed by an expert neurologist (DMG or TBM); parameters for EEG analysis were EEG abnormalities with epileptic meaning (EA) and EEG abnormalities without specific epileptic meaning (non-EA). “EA” included generalized and focal seizures, status epilepticus (SE), generalized periodic discharges (GPDs) and lateralized periodic discharges (LPDs); “Non-EA” included focal or diffuse slow wave activity, sharp waves, EEG asymmetries in frequency or amplitude.

Length of monitoring was decided according with clinical indication.

Data were shown as mean ± standard deviation. T-test for unpaired data was used as appropriate. *p* < 0.01 was considered as statistically significant.

## Results

In both groups cEEG was obtained in all cases. Demographic data are shown in Table 1. Indication for cEEG was seizure detection in comatose patients in all cases. The EEG montage included 8 scalp electrodes in both the patient and the control group.

**Table 1:** Demographic data of the study groups. Data are expressed as mean ± SD. No statistical difference between the groups was observed

	Study group *n* = 20	Control group n = 20
*Age*	58.5 ± 12.1	63.9 ± 15.0
*Gender*		
*Male*	8	11
*Female*	12	9
*Diagnosis*		
*SAH*	5	8
*ICH*	15	12
*ICU LOS*	7.3 ± 3.7	8.6 ± 3.1
*Surgical patients*	12	9
*GOS on ICU dimission*	4.1 ± 0.6	4.0 ± 0.7

Main results are shown in Table 2. Time for electrodes positioning was significantly shorter in Study group (*p* < 0.0001). The length of monitoring was longer in Control group; nevertheless in Study group it was longer than 24 h in 13 cases (43% of patients). During this time, 35 interventions were necessary to obtain a good quality EEG tracing in Study group and 11 in Control group; (*p* < 0.01) (Table 2). Interventions corrected the technical problem in all cases.

**Table 2:** Main results are shown. Data are expressed as mean ± SD. *p* < 0.01 ** was considered as statistical significance

	Study group *n* = 20	Control group *n* = 20
*Time to assembly (min)*	6.2 ± 1.1	10.4 ± 2.3**
*Length of monitoring (hrs)*	57 ± 12	75 ± 15 **
*Interventions (n)*	35	11
*Interventions per patient (n)*	1.7 ± 1.2	0.5 ± 0.6**
*Electrodes replacement*	4	3
*Gel or paste application*	31	8
***Epileptic abnormalities***		
*Status epilepticus*	3	2
*GPDs*	3	2
*LPDs*	1	1
***Non epileptic abnormalities***		
*Focal slow waves*	1	3
*General slow waves*	4	3
*EEG Asymmetries*	2	3

EEG abnormalities were often recorded; EA and no EA were respectively recorded in 7 cases (35%) and in 7 cases (35%) in Study Group, and in 5 cases (25%) and in 9 cases (45%) in Control Group. (*p* > 0.05) (Table 2). EEG led to anti-seizure medications in 10 cases in Study Group and in 7 cases in Control Group.

In Control group no cutaneous lesions were observed after electrodes removal; in Study group 17 patients showed pressure lesions, that consisted in skin redness. They appeared after a mean time of 15 ± 2 h and spontaneously recovered with no intervention. In 4 cases, the risk of more serious lesions led us to stop EEG monitoring. This occurred after a mean time of 52 ± 4 h. After EEG interruption, no skin lesion was observed in any patient.

In no case EEG technician intervention was required in Study group.

## Discussion

According with this single-center feasibility study, EEG helmet CerebAir® was simple and quick to apply, and was used for continuous recordings lasting more than 24 h; it was positioned by a neuro ICU physician and provided good quality cEEG without the need of EEG technician. When used for continuous monitoring, skin should be frequently checked, and lesions must be prevented.

cEEG is frequently used in Intensive Care Units, and its use is much wider than a few years ago [4]. Several studies have shown that using conventional 20 min-EEG recording many unrecognized EEG abnormalities can be present [5, 6]. This is particularly true in patients who remain unconscious after a seizure, or in patients in coma without a clear interpretation. Recently, both the European Society of Intensive Care [1] and the American Society of Clinical Neurophysiology [2] recommended this technique in many conditions. Even if how long cEEG should last is not known, the probability of abnormalities detection during cEEG increases with the duration of monitoring, and 24–48 h of recording was considered as reasonable.

Basing on these recommendations, a greater availability of these methods in the hospital is desirable, and the absence of a neurophysiology service 24/7 may be a limiting factor. For this reason, easy-to-use systems may be an interesting option in emergency settings as in Intensive care Unit. If they may have a role in these conditions is still not known.

The system we used was found to be quicker if compared with simplified conventional recording. This is in part due to the features of the helmet, that is rigid but adjustable by belts on the scalp of the patient. Furthermore, it has fixed positions for electrodes, that are connected with helmets by metallic clips. They are made by a plastic structure filled with conductive gel; in this way, they may adhere to the skin of the patient even in difficult technical conditions, eliminating the need of skin preparation.

Moreover, wireless system is a useful feature in Intensive Care Unit, where several machines are needed at bedside, and nursing procedures may limit the quality of EEG tracing.

It enabled a continuous recording of EEG signal for an extended period, up to 3 days in our case series; after recording, pressure lesions were frequently observed, but consisted only in skin redness. This could be a problem in a larger population. In our case series, prevention of skin lesions led to interruption of the study in 4 cases; this occurred in all cases after at least 36 h of monitoring. Actually, this device was designed for quick diagnosis in an emergency setting, and not for continuous monitoring. We showed that it can perform EEG for more than 24 h; in these cases, as recommended by manufacturer, skin should be frequently checked. In our experience adding gel on electrodes and adjusting the helmet frequently could be interesting options to reduce the risks and to increase length of monitoring.

Even if a higher number of interventions were necessary to correct artifacts in comparison with conventional recording, electrodes impedance was optimal for the most time of the monitoring; due to features of hydrogel electrodes, artifacts were generally corrected by applying a supplementary conductive gel on the skin.

Cleaning and disinfection were easy and not time consuming both for the helmet, and for the fastening belts. Replacing batteries daily was always necessary.

In our opinion, the availability of technology that may obtain a quick EEG acquisition in Intensive Care Unit is an important result. An important step forward in this process was obtained by disposable hydrogel EEG electrodes, that eliminated the need of skin preparation. Ziai, using a commercially available EEG cap with hydrogel electrodes installed by EEG technician, obtained a reliable EEG tracing in Emergency Department that helped to clinical diagnosis [7]. Furthermore, several authors tried to simplify EEG positioning, reducing the number of electrodes or using hairline montage. Results are controversial.

Most of the studies used hairline montage, showing unacceptably poor sensitivity for seizure detection (60–70%) but rather good specificity (> 90%) [3, 6, 8]. Vanherpe observed that 8-lead montage proved to be reliable for the detection of electrographic seizure activity in a post anoxic population, but diagnostic accuracy was low by using hairline montage [9].

Karakis found a sensitivity for seizure detection was 92.5%, and a specificity of 93.5% by using a 7- electrodes non-hairline positioning, suggesting that it could potentially be a quick and reliable EEG montage for seizures detection in the intensive care unit [10]. Meyer and Egawa used CerebAir® for continuous EEG monitoring and found high accuracy in detecting EEG abnormalities [11, 12].

Others authors found very low accuracy by using devices designed for different aims [13]. Some reports investigated depth of anesthesia monitors such as BIS or Entropy in ICU [14]. They are based on three or four frontal electrodes, and are recommended to reduce drug consumption and risk of awareness during anesthesia [15]. They could have a role in ICU for monitoring burst suppression, but are not designed for seizures diagnosis. Data are still insufficient to draw any conclusion on this topic [16].

Even if our study was not powered to this aim, we found that incidence of EEG abnormalities was similar in two groups and is comparable to previous data [5]; methodology of the study prevents us from drawing any conclusion regarding a direct performance comparison. In fact, recording was done on two different patient groups, and it is unknown if missed seizures or false positive can be occurred.

Moreover, this study has further limitations.

Number of patients was low. Forty cases were sufficient to validate its feasibility in emergency settings, but we cannot draw conclusions about accuracy of the system for seizure diagnosis in comparison with conventional EEG. In particular, the reduced number of electrodes is very practical for a quick montage but precludes accuracy in difficult EEG diagnosis.

Furthermore, we considered surgical dressings as exclusion criterion. This may be an important bias, since post-operative patients are often candidates to EEG monitoring. In addition, risk of infections could be higher if electrodes positioned very close to surgical dressing. Clinicians should keep in mind that rigid headset cannot be considered in these situations.

## Conclusions

Even if with these limitations, in this feasibility study we found that a good quality EEG tracing was easy to obtain by this device, even if positioned by ICU physician, and EEG technician was not mandatory. It was faster to position if compared with standard techniques, and can be used for brief periods of continuous EEG monitoring. It could be very useful as part of diagnostic process in an emergency setting to rule out non convulsive seizures when cause of coma or of neurological deterioration is not clearly defined, and standard EEG is not available.

Recently, several studies observed that after a relatively short education, ICU nurses and doctors can reach an acceptable level of expertise to identify the main EEG patterns and to solve technical problems of recording when neurologist is not available [17, 18]. This is an interesting challenge for neurointensivist [19]. EEG systems like CerebAir® can facilitate this approach, giving to the Intensive Care physician an additional instrument to improve the care of patients with consciousness disorders.

## Data Availability

The datasets used and/or analyzed during the current study are available from the corresponding author on reasonable request.

## Abbreviations

EEG: Electroencephalogram
cEEG: Continuous Electroencephalogram
ICU: Intensive Care Unit
EA: Epileptic abnormalities
non-EA: Non epileptic Abnormalities
SE: Status epilepticus
GPDs: Generalized periodic discharges
LPDs: Lateralized periodic discharges
DMG: Della Marca Giacomo
TBM: Tiziana Benzi Markushi
SAH: Subarachnoid hemorrhage
ICH: Intracerebral hemorrhage
LOS: Length of staying
GOS: Glasgow Outcome Scale
